# Supplementary material for: JNK pathway restricts DENV2, ZIKV and CHIKV infection by activating complement and apoptosis in mosquito salivary glands
Source: PLoS Pathog. 2020 Aug 10;16(8):e1008754. doi: 10.1371/journal.ppat.1008754 (PMC7444518; doi:10.1371/journal.ppat.1008754)
Supplement: S1 Text — (DOCX) [file ppat.1008754.s001.docx]

**Supplementary Information**

**S1 Text**

**Common DEGs among DENV2, ZIKV and CHIKV**

Our comparative high throughput RNA-seq data revealed 19 DEGs common to DENV2, ZIKV or CHIKV infection in *A. aegypti* SGs. They included six commonly upregulated, 11 commonly downregulated and two differentially-regulated DEGs. Commonly upregulated genes included the transcription termination factor *Lodestar* (AAEL018118) (1), *Ribunucleoside disphosphate reductase* (AAEL010691) that is involved in DNA synthesis, two immune genes, namely *Juvenile Hormone inducible (JHI,* AAEL000515*)* and *Dicer2 (Dcr2,* AAEL006794*)*, one oxidative stress responsive gene *Cytochrome6Z9* (AAEL009129), and one gene of unknown function (AAEL003732/EAT44971). DEGs that were commonly downregulated included a digestive enzyme, *MaltaseA1* (AAEL009524), three blood-feeding facilitating genes, namely an *apyrase* (AAEL006347), a *34kDa family secreted protein* (AAEL003600) and a *salivary* *mucin4* (AAEL003100), *Trypsin3A1* (AAEL007818), one stress-responsive gene *Cytochrome9J26* (AAEL014609), one antimicrobial peptide, *Gambicin1* (AAEL004522), a *venom allergen* (AAEL000793) and three genes of unknown functions (AAEL009081, AAEL004899, AAEL010242). The CLIP-domain serine protease *CLIPB37* (AAEL005431) and a fibrinogen-related pathogen pattern recognition protein (AAEL008646) were upregulated by ZIKV and CHIKV, and downregulated by DENV2. The largest proportion of DEGs was virus-specific. Although the difference in collection time between the flaviviruses and alphavirus could play a role, these transcriptome signatures reflect virus-specific regulation in SGs.

**DEGs related to immune effectors**

AMPs are the hallmark of immune activation and can be regulated independently or in combination by different pathways (Fig. 1A; S6 and 7 Fig) (2–4). Strikingly, *Gambicin1* (*Gam1*) was downregulated by the three viruses (Fig. 1C; S1 Table), indicating immune inhibition. However, Gam1 shows no effect on DENV2 replication (5). Defensins (*DefA* and *DefD*) and cecropins (*CecA*, *CecB* and *CecG*) that have been reported to antagonize arboviruses (6,7) were upregulated upon ZIKV infection. On the contrary, upon DENV2 infection *CecG* was downregulated. DENV2 inhibition of *CecG* expression was associated to sfRNA in SGs (8). It would be interesting to test whether the discrepancy in *CecG* expression between DENV2 and ZIKV is due to variation in sfRNA function.

The complement system can eliminate pathogens by opsonization, phagocytosis or lysis (9) and is regulated by different immune pathways. Leucine-rich repeat (LRR) proteins guide the binding of TEPs to pathogens and activate the complement system (10). In *A. aegypti*, TEP15 mediates an anti-DENV2 response (11). We observed the upregulation of several TEPs, including *TEP15*, by ZIKV and CHIKV. However, 17 LRRs showed virus-specific differential regulation (Fig 1; S7 Fig; S1 Table), suggesting virus-specific variation in complement activation in SGs.

**DEGs related to apoptosis**

In *A. aegypti*, the core apoptotic pathway is activated by Dronc and Ark, which are repressed by Inhibitor of Apoptosis Protein1 (IAP1) (12,13). Upon activation, Dronc cleaves effector caspases, such as Caspase8, which then degrade physiological substrates, leading to apoptosis (13). Upstream of the core pathway, IAP1 is inhibited by a set of proteins including Head involution (Hid). *Hid* transcription is induced by p53 (14) and Forkhead box O (FoxO)(15), the latter being activated by Protein kinase C53E (Pkc53E) (16,17). Alternatively, caspase-independent apoptosis is initiated by death executioner bcl2 (Debcl2) (18). A DENV2 refractory strain of *A. aegypti* showed higher *Dronc* expression in the midgut, and silencing of the gene significantly increased the susceptibility of this strain to DENV2 infection (19). Interestingly, *Dronc* silencing reduced midgut infection of *A. aegypti* when challenged with Sindbis virus (Alphavirus) (20). This suggests that infection depends on a fine regulation of apoptosis.

We found 27 DEGs related to apoptosis and autophagy in DENV2 or CHIKV infection (S1 Table). Some pro-apoptotic factors like *Caspase8* (AAEL014348) and *p53* (AAEL007595) were upregulated, while other pro-apoptotic factors such as *Pkc53E* (AAEL001108), *FoxO* (AAEL012847) and *Debcl2* (AAEL001515) were downregulated by DENV2 or CHIKV infection. Pro-apoptotic factors, *Ark* (AAEL000874) and *Dronc* (AAEL011562), and anti-apoptotic factor *IAP1* (AAEL009074) were upregulated only by CHIKV. Such contrasting regulatory mechanisms may be triggered due to vector-virus counteractive interactions, where the vector induces apoptosis as a defense against arbovirus, while viruses may block the apoptotic signaling (21). Autophagy, which is reported to be a pro-viral phenomenon, intertwines with apoptosis (22,23). Two autophagy related genes (*ATG14*, AAEL001133; *ATG18A*, AAEL013063) were upregulated upon CHIKV infection. Thus, apoptosis is tightly-regulated upon arbovirus infection in SGs.

**DEGs related to blood-feeding**

Arboviruses are transmitted by mosquito saliva secretion during skin probing (24–26). Alteration of salivary components can alter blood feeding, and thus enhance transmission by: i) increasing probing attempts and probing time (27); ii) reducing the production of salivary proteins that directly inhibit virus infection (28); and iii) enhancing amounts of allergens or other inflammatory compounds that increase infection (29). We noted that DENV2, ZIKV and CHIKV modulated 13, 13 and 27 DEGs related to blood feeding, respectively (S1 Table). Interestingly, four of these DEGs were commonly downregulated by all three viruses. These included an *apyrase* (*ATP diphosphohydrolase*) (AAEL006347), a *salivary mucin* (AAEL003100), a *34 kDa protein* (AAEL003600) and a putative *salivary secreted peptide gene* (AAEL004899). Two other apyrases (AAEL000575 and AAEL006333) were downregulated by DENV2 and ZIKV, respectively, while nine other mucins were differentially regulated by the three viruses (S1 Table). Apyrases hydrolyze ATP and ADP to AMP, preventing platelet aggregation during blood-feeding(Dong et al., 2012; Sim et al., 2012; Sun et al., 2006). Apyrase contents in salivary gland extracts from various mosquito species inversely correlated to probing attempts (30). Mucins may play a role in lubricating the insertion of mouthparts and influence probing. The 34 kDa protein has been shown to enhance DENV2 infection of keratinocytes by inhibiting the interferon response (31), and the role of the putative salivary secreted peptide, an orthologue of a tick saliva protein (32), is unknown. Five D7 family genes were downregulated (AAEL006417, AAEL007394, AAEL006423, AAEL006424, AAEL002726) upon ZIKV or CHIKV infection. These highly abundant mosquito saliva proteins (33–35) scavenge biogenic amines (36) and reduce vasoconstriction, platelet-aggregating and pain-inducing properties (36–38). One of these proteins (AAEL006424) inhibits DENV2 infection in vertebrates (28).

Three odorant binding proteins (OBPs; AAEL018102, AAEL002587 and AAEL000124) were downregulated by DENV2 or CHIKV, while another one (AAEL005770) was upregulated by ZIKV. DENV2-infected SGs showed increased expression of two other OBPs that are involved in probing initiation (27). OBPs bind to hydrophobic odorant chemicals and carry them to odorant receptors, thereby influencing olfactory and gustatory signaling (39).

**DEGs related to lipid metabolism**

Enveloped viruses such as DENV2, ZIKV and CHIKV interact with host lipid membranes for entry, replication, translation, assembly and egress(40,41). Flavivirus infection alters lipid profile in mosquitoes (42,43) and is restricted by lipid biogenesis inhibition in mosquito cells (44). We found 22 DEGs related to lipid metabolism, indicating a strong alteration of the lipid profile in SGs (S1 Table). Two homologues of *fatty acid synthase* (*FAS*) (AAEL002228 and AAEL001194), which initiate fatty acid biogenesis, were downregulated by ZIKV and CHIKV infection. On the contrary, genes involved downstream of FAS, such as *elongases* (AAEL009574, AAEL013128), *desaturase* (AAEL003645) and *reductase* (AAEL006774), were upregulated by CHIKV. Enzymes involved in synthesis of phospholipids and sphingolipids were also altered. *Glycerol-3-phosphate acyltransferase* (AAEL012743) that initiates phospholipid biogenesis was downregulated by ZIKV. Two *phospholipase A2s* (AAEL001523, AAEL001528) that hydrolyze the 2-acyl ester, and one *phospholipase D* (AAEL000264) that hydrolyses the basic head group were upregulated by CHIKV. *Sphingomyelin synthetases* (AAEL004710, AAEL001381) were upregulated by CHIKV, while *sphingolipid phospholipase* (AAEL003402) was downregulated upon DENV2 infection. Interestingly, the gene associated with lipid droplets (AAEL005951), induced by DENV2 infection in mosquito Aag2 cells (45), was downregulated by both DENV2 and CHIKV in mosquito SGs.

**Supplementary References**

1. Girdham CH, Glover DM. Chromosome tangling and breakage at anaphase result from mutations in lodestar, a *Drosophila* gene encoding a putative nucleoside triphosphate-binding protein. Genes Dev. 1991;5(10):1786–99.

2. Ramirez JL, Dimopoulos G. The Toll immune signaling pathway control conserved anti-dengue defenses across diverse *Ae. aegypti* strains and against multiple dengue virus serotypes. Dev Comp Immunol. 2010;34(6):625–9.

3. Wang S, Beerntsen BT. Functional implications of the peptidoglycan recognition proteins in the immunity of the yellow fever mosquito, *Aedes aegypti*. Insect Mol Biol. 2015;24(3):293–310.

4. Zhang R, Zhu Y, Pang X, Xiao X, Zhang R, Cheng G. Regulation of antimicrobial peptides in *Aedes aegypti* Aag2 Cells. Front cell infect microbiol. 2017;7.

5. Jupatanakul N, Sim S, Angleró-Rodríguez YI, Souza-Neto J, Das S, Poti KE, et al. Engineered *Aedes aegypti* JAK/STAT pathway-mediated immunity to dengue virus. PLoS Negl Trop Dis. 2017;11(1).

6. Luplertlop N, Surasombatpattana P, Patramool S, Dumas E, Wasinpiyamongkol L, Saune L, et al. Induction of a peptide with activity against a broad spectrum of pathogens in the *Aedes aegypti* salivary gland, following infection with dengue virus. PLoS Pathog. 2011 ;7(1):e1001252.

7. Xiao X, Liu Y, Zhang X, Wang J, Li Z, Pang X, et al. Complement-related proteins control the flavivirus infection of *Aedes aegypti* by inducing antimicrobial peptides. PLoS Pathog. 2014;10(4).

8. Pompon J, Manuel M, Ng GK, Wong B, Shan C, Manokaran G, et al. Dengue subgenomic flaviviral RNA disrupts immunity in mosquito salivary glands to increase virus transmission.PLOS Pathog. 2017;13(7):e1006535.

9. Shishido SN, Varahan S, Yuan K, Li X, Fleming SD. Humoral innate immune response and disease. Clin Immunol. 2012;144(2):142–58.

10. Fraiture M, Baxter RHG, Steinert S, Chelliah Y, Frolet C, Quispe-Tintaya W, et al. Two mosquito LRR proteins function as complement control factors in the TEP1-mediated killing of *Plasmodium*. Cell Host Microbe. 2009;5(3):273–84.

11. Cheng G, Liu L, Wang P, Zhang Y, Zhao YO, Colpitts TM, et al. An *in vivo* transfection approach elucidates a role for *Aedes aegypti* thioester-containing proteins in flaviviral infection. PLoS One. 2011;6(7):e22786.

12. Clarke TE, Clem RJ. Insect defenses against virus infection: the role of apoptosis. Int Rev Immunol. 2003;22(5–6):401–24.

13. Liu Q, Clem RJ. Defining the core apoptosis pathway in the mosquito disease vector *Aedes aegypti*: the roles of iap1, ark, dronc, and effector caspases. Apoptosis. 2011;16(2):105–13.

14. Mollereau B, Ma D. The p53 control of apoptosis and proliferation: lessons from Drosophila. Apoptosis. 2014;19(10):1421–9.

15. Luo X, Puig O, Hyun J, Bohmann D, Jasper H. Foxo and Fos regulate the decision between cell death and survival in response to UV irradiation. EMBO J. 2007;26(2):380–90.

16. Mattila J, Kallijärvi J, Puig O. RNAi screening for kinases and phosphatases identifies FoxO regulators. Proc Natl Acad Sci U S A. 2008;105(39):14873–8.

17. Puig O, Marr MT, Ruhf ML, Tjian R. Control of cell number by *Drosophila* FOXO: downstream and feedback regulation of the insulin receptor pathway. Genes Dev. 2003;17(16):2006–20.

18. Galindo KA, Lu W-J, Park JH, Abrams JM. The Bax/Bak ortholog in *Drosophila*, Debcl, exerts limited control over programmed cell death. Development. 2009;136(2):275–83.

19. Ocampo CB, Caicedo PA, Jaramillo G, Ursic Bedoya R, Baron O, Serrato IM, et al. Differential expression of apoptosis related genes in selected strains of *Aedes aegypti* with different susceptibilities to dengue virus. PLoS One. 2013;8(4):e61187.

20. Wang H, Gort T, Boyle DL, Clem RJ. Effects of manipulating apoptosis on sindbis virus infection of *Aedes aegypti* mosquitoes. J Virol. 2012;86(12):6546–54.

21. Shaoli Lin, Yan-Jin Zhang. Interference of apoptosis by hepatitis B virus. Viruses. 2017;9(8):230.

22. Brackney DE. Implications of autophagy on arbovirus infection of mosquitoes. Curr Opin Insect Sci. 2017;22:1–6.

23. Maiuri MC, Zalckvar E, Kimchi A, Kroemer G. Self-eating and self-killing: crosstalk between autophagy and apoptosis. Nat Rev Mol Cell Biol. 2007;8(9):741–52.

24. Choumet V, Attout T, Chartier L, Khun H, Sautereau J, Robbe-Vincent A, et al. Visualizing non infectious and infectious anopheles gambiae blood feedings in naive and saliva-immunized mice. PLoS One. 2012;7(12):e50464.

25. Duangkhae P, Erdos G, Ryman KD, Watkins SC, Falo LD, Marques ETA, et al. Interplay between keratinocytes and myeloid cells drives dengue virus spread in human skin. J Invest Dermatol. 2018;138(3):618–26.

26. Styer LM, Kent KA, Albright RG, Bennett CJ, Kramer LD, Bernard KA. Mosquitoes inoculate high doses of West Nile virus as they probe and feed on live hosts. PLoS Pathog. 2007;3(9):e132.

27. Sim S, Ramirez JL, Dimopoulos G. Dengue virus infection of the *Aedes aegypti* salivary gland and chemosensory apparatus induces genes that modulate infection and blood-feeding behavior. PLoS Pathog. 2012;8(3):e1002631.

28. Conway MJ, Londono-Renteria B, Troupin A, Watson AM, Klimstra WB, Fikrig E, et al. *Aedes aegypti* D7 saliva protein inhibits dengue virus infection. PLoS Negl Trop Dis. 2016;10(9):e0004941.

29. Schmid MA, Glasner DR, Shah S, Michlmayr D, Kramer LD, Harris E. Mosquito saliva increases endothelial permeability in the skin, immune cell migration, and dengue pathogenesis during antibody-dependent enhancement. PLoS Pathog. 2016;12(6):e1005676.

30. Ribeiro JMC, Rossignol PA, Spielman A. Salivary gland apyrase determines probing time in anopheline mosquitoes. J Insect Physiol. 1985;31(9):689–92.

31. Surasombatpattana P, Ekchariyawat P, Hamel R, Patramool S, Thongrungkiat S, Denizot M, et al. *Aedes aegypti* saliva contains a prominent 34-kDa protein that strongly enhances dengue virus replication in human keratinocytes. J Invest Dermatol. 2014;134(1):281–4.

32. Francischetti IMB, Meng Z, Mans BJ, Gudderra N, Hall M, Veenstra TD, et al. An insight into the salivary transcriptome and proteome of the soft tick and vector of epizootic bovine abortion, *Ornithodoros coriaceus*. J Proteomics. 2008;71(5):493–512.

33. Arcà B, Lombardo F, Francischetti IMB, Pham VM, Mestres-Simon M, Andersen JF, et al. An insight into the sialome of the adult female mosquito *Aedes albopictus*. Insect Biochem Mol Biol. 2007;37(2):107–27.

34. Fontaine A, Fusaï T, Briolant S, Buffet S, Villard C, Baudelet E, et al. *Anopheles* salivary gland proteomes from major malaria vectors. BMC Genomics. 2012;13(1):614.

35. Ribeiro JMC, Martin-Martin I, Arcà B, Calvo E. A deep insight into the sialome of male and female *Aedes aegypti* mosquitoes. PLoS One. 2016;11(3):e0151400.

36. Calvo E, Mans BJ, Andersen JF, Ribeiro JMC. Function and evolution of a mosquito salivary protein family. J Biol Chem. 2006;281(4):1935–42.

37. Andersen JF, Francischetti IMB, Valenzuela JG, Schuck P, Ribeiro JMC. Inhibition of hemostasis by a high affinity biogenic amine-binding protein from the saliva of a blood-feeding Insect. J Biol Chem. 2003;278(7):4611–7.

38. Sangamnatdej S, Paesen GC, Slovak M, Nuttall PA. A high affinity serotonin- and histamine-binding lipocalin from tick saliva. Insect Mol Biol. 2002;11(1):79–86.

39. Pelosi P, Zhou J-J, Ban LP, Calvello M. Soluble proteins in insect chemical communication. Cell Mol Life Sci. 2006;63(14):1658–76.

40. Martín-Acebes MA, Vázquez-Calvo Á, Saiz J-C. Lipids and flaviviruses, present and future perspectives for the control of dengue, Zika, and West Nile viruses. Prog Lipid Res. 2016 ;64:123–37.

41. Villareal VA, Rodgers MA, Costello DA, Yang PL. Targeting host lipid synthesis and metabolism to inhibit dengue and hepatitis C viruses. Antiviral Res. 2015;124:110–21.

42. Chotiwan N, Andre BG, Sanchez-Vargas I, Islam MN, Grabowski JM, Hopf-Jannasch A, et al. Dynamic remodeling of lipids coincides with dengue virus replication in the midgut of *Aedes aegypti* mosquitoes. PLoS Pathog. 2018;14(2).

43. Vial T, Tan W-L, Wong Wei Xiang B, Missé D, Deharo E, Marti G, et al. Dengue virus reduces AGPAT1 expression to alter phospholipids and enhance infection in *Aedes aegypti*. PLoS Pathog. 2019;15(12):e1008199.

44. Martín-Acebes MA, Blázquez A-B, Jiménez de Oya N, Escribano-Romero E, Saiz J-C. West Nile virus replication requires fatty acid synthesis but is independent on phosphatidylinositol-4-phosphate lipids. PLoS One. 2011;6(9).

45. Barletta ABF, Alves LR, Nascimento Silva MCL, Sim S, Dimopoulos G, Liechocki S, et al. Emerging role of lipid droplets in *Aedes aegypti* immune response against bacteria and dengue virus. Sci Rep. 2016;6(1).

46. Bonizzoni M, Dunn WA, Campbell CL, Olson KE, Marinotti O, James AA. Complex modulation of the *Aedes aegypti* transcriptome in response to dengue virus infection. PLoS One. 2012;7(11):e50512.

**Supplementary Tables**

**S1 Table. Fold changes and functional annotations for DEGs in SGs upon infection with DENV2, ZIKV and CHIKV. (Excel sheet)**

**S2 Table. Primer sequences for candidate gene RNAi silencing and RT-qPCR quantification**

**S3 Table. Primer sequences for DEG validation**

**S4 Table. Primer sequences for JNK pathway-controlled gene expressions and corresponding RNAi silencing**

**S5 Table. Primer sequences for virus quantification**
