# Supplementary material for: JNK pathway restricts DENV2, ZIKV and CHIKV infection by activating complement and apoptosis in mosquito salivary glands
Source: PLoS Pathog. 2020 Aug 10;16(8):e1008754. doi: 10.1371/journal.ppat.1008754 (PMC7444518; doi:10.1371/journal.ppat.1008754)
Supplement: S2 Table — (DOCX) [file ppat.1008754.s003.docx]

**S2 Table.**

| **Gene name/ Acc. No.** | **dsRNA primers** | **RT-qPCR primers** |
| --- | --- | --- |
| *Actin* / AAEL011197 |  | Fw: GAACACCCAGTCCTGCTGACA  Rv: TGCGTCATCTTCTCACGGTTAG |
| *Lac Z* | Fw: TACCCGTAGGTAGTCACGCA  Rv: TACGATGCGCCCATCTACAC |  |
| *Kayak* / AAEL008953 | Fw: GCCTCCTTTGACGGCTTAC  Rv: CTGCGCTACGGCTACGTC | Fw: CCTCACCGATAGCTTGGACA Rv: GCGGTAGATTCACACTGGTC |
| *Snake-like* / AAEL002273 | Fw: GTGGTGCGTTTGGGAGAGTA  Rv: GCACAGATCTGCGAATCAAT | Fw: CATGTCGATCGTCCACGAAG  Rv: ACTCACCTTCCAGAGCTTCC |
|  |  |  |
| *Easter-like* / AAEL012775 | Fw: CGCCATATTCGAGTTCCCT  Rv: CAACCCAATGTCGTTGGAA | Fw: AATAGTGCTTGGGTGCTTGC Rv: CGGTGTGGTACAGGATGGAT |
| *CLIPB21* / AAEL001084 | Fw: AGGACGGCTCGGAGAAGTA  Rv: GATTCCTCGTTCAAACAGGCT | Fw: GGTGGAGGACTGATGGTTCA Rv: TTCCAGTAGGCCGTGACATT |
| *CLIPB13A* / AAEL003243 | Fw: CAGTGCGTACTCCGAGGTG  Rv: GCAGCCCTTTTGATGTAATTG | Fw: ATAGAACGAGACTGCGCTGA Rv: GTCGAACGTTTGCCTCTGAA |
| *IKK2* / AAEL012510 | Fw: AGTGTCATCATGGGCGAAAC  Rv: ACGTTTGTCCTGTTCTGCG | Fw: CGTGGCGAAGAATTTGGAGT Rv: AGGGTGTTCAGTTCACGGAT |
| *Gale5* / AAEL003844 | Fw: ATCCGCATCAACCAAGCTAC  Rv: GCTGCTGTGGAGGGTTGTTA | Fw: GGTGGCTGTGTGATCCATTC Rv: GGTACATATGGTGGCGGTTG |
| *Juvenile Hormone Inducible* / AAEL000515 | Fw: ACCTTGATGGCCGAGAGAAT  Rv: CCTGCCATTTTCGTATTTGA | Fw: TTATGAAAGGCGACGAAGCG Rv: TCTCGGACACCAATCTTCCC |
| *Ectoderm Expressed4* / AAEL014931 | Fw: GCAATTGCTCAGGCACAAC  Rv: GCCATCGGCATCTTTTCTT | Fw: AAGTGCCACACAAACTGTCC Rv: CCCATTCTCGTACGTCCTCA |
