# Supplementary material for: JNK pathway restricts DENV2, ZIKV and CHIKV infection by activating complement and apoptosis in mosquito salivary glands
Source: PLoS Pathog. 2020 Aug 10;16(8):e1008754. doi: 10.1371/journal.ppat.1008754 (PMC7444518; doi:10.1371/journal.ppat.1008754)
Supplement: S3 Table — (DOCX) [file ppat.1008754.s004.docx]

**S3 Table.**

| **Virus infection** | **Gene Accession No.** | **Primer sequences** |
| --- | --- | --- |
| DENV | AAEL017469 | Fw: ACTACTGTGCCGGATTCTGT  Rv: ATTGGACTGAACAGCAGAGC |
|  | AAEL000080 | Fw: TCTCCGACAACTCGGATTTCA Rv: TTCTTGCCGAGAAGGGAGTT |
|  | AAEL006704 | Fw: CATCTGGGTTGGTACTCCGA Rv: TCTCGTCAGAACGATACGTGA |
|  | AAEL006953 | Fw: CCTGCTGCTGAAGATCAACC Rv: CGGAGTTTCCATTGGAGCTG |
|  | AAEL003844 | Fw: CAGGCTATCCCATTGGCAAC Rv: GTGGGTAGCTTGGTTGATGC |
|  | AAEL012441 | Fw: TGGATGTGGAGATTGCCGAT Rv: GGTTGTGCCACACGTTAGTT |
|  | AAEL007191 | Fw: GGTGTGATCTTGCCTCTGGA Rv: AGCACCATGGCTTTGTTGAG |
|  | AAEL003203 | Fw: CGGTTTCGCATTCCTGCTAT Rv: AACACGCACAGAATGATCCG |
|  | AAEL015121 | Fw: TGCACGCTTTCAGAAACTCC Rv: TTTGAGAACTCGCAGTGGGT |
|  | AAEL006123 | Fw: ATGGAGCCATCACCATCGAA Rv: GAGTGCACAATCGAAGGCAT |
|  | AAEL006498 | Fw: CGCGATGTTTGGATCACTGT Rv: CAAGGCACCATTGTTGGTCA |
|  | AAEL006259 | Fw: CTCCAACCTGCTAGTGGTCA Rv: CCAAGGCAAGCGTAGACTTC |
| ZIKV | AAEL001673 | Fw: CTTCTCTTCCGGTGAAAGGC  Rv: CCGGATCCGTTATCAACGAC |
|  | AAEL002759 | Fw: CTGATGAAGTGTCCCGCAAG Rv: ACCGACGACCTTCAACTCTT |
|  | AAEL006704 | Fw: CATCTGGGTTGGTACTCCGA Rv: TCTCGTCAGAACGATACGTGA |
|  | AAEL008283 | Fw: ATGGACGACCTCTCAGTTGG Rv: TGGGATGATCACATGCCAGT |
|  | AAEL004783 | Fw: TGGTGGTGATGTCCCACAAT Rv: TCGGTGTCGTCATAATCGGT |
|  | AAEL014937 | Fw: AGCCTTTGCATTTCCACCTG Rv: CAGACGACTTGTGGTTAGCG |
|  | AAEL004382 | Fw: GCGTTCAAGCTCCGTAATCA Rv: CCAGCGATGGGATCAGGAAA |
|  | AAEL000886 | Fw: AACTCCTGGCCATCGTAGTC Rv: CACCACATTTCGAAGCCAGT |
|  | AAEL005676 | Fw: AATTTGATTGACGCCGTCCT Rv: GGCAGTCATCCACCAGTTTC |
|  | AAEL000647 | Fw: GAGCGAAGAAGAGGTTACGC Rv: CACGGCTGAACTTCTAAGCA |
|  | AAEL007818 | Fw: AGTTTACCGGCTACCGCATA Rv: GCTGAAACTTGGCTGAGTCC |
| CHIKV | AAEL003596 | Fw: TGGACGTGGAAGTTGATGGA  Rv: CGTGGAAGGTAATCTCGATGC |
|  | AAEL005331 | Fw: TAGCGCACTCTTTGTTCACG Rv: GCGATTCTGCTGGACAAGTT |
|  | AAEL006050 | Fw: AAGTGGCTGATCCGTTTGTG Rv: CGTCGTGTCAAAGCCATTCT |
|  | AAEL003118 | Fw: AAGCCTCACCAGCATCAGAA Rv: TGTCACTCTTGGCAACAAGC |
|  | AAEL013341 | Fw: ATATGTTGCCAAGCGGTCAC Rv: GAAGCGATTTCTTCGGTGCT |
|  | AAEL006129 | Fw: CCATACGCATCCGTTGATCC Rv: CGGTTGAACAGCGACTCATT |
|  | AAEL008646 | Fw: AGTTTAAGGCACGAGGACCA Rv: CCGAACTTGGTTTGCTCACA |
|  | AAEL001627 | Fw: TTCCCACTGGATGGAAGCAT Rv: TAGCAATCGTCGCTGATCCT |
|  | AAEL001169 | Fw: GCTGACGGATGAAGACATCG Rv: GCTGACGGATGAAGACATCG |
|  | AAEL011499 | Fw: GCTTTGATTGACCCACCGTT Rv: CAGCTTGGTTCCCTCCATTG |
|  | AAEL002661 | Fw: GGAAGACGGTTCGTGTTCAG Rv: TCTCTCCGCACATCTCCATC |
|  | AAEL009750 | Fw: TCTACGACTATGGTGACTGCT Rv: CGATGCTGGTTCGGAATTGT |
|  | AAEL003060 | Fw: CCGTCATTCGTGTGGACAAA Rv: GGAAGCACGACTTTCCTCAG |
