# Supplementary material for: JNK pathway restricts DENV2, ZIKV and CHIKV infection by activating complement and apoptosis in mosquito salivary glands
Source: PLoS Pathog. 2020 Aug 10;16(8):e1008754. doi: 10.1371/journal.ppat.1008754 (PMC7444518; doi:10.1371/journal.ppat.1008754)
Supplement: S4 Table — (DOCX) [file ppat.1008754.s005.docx]

**S4 Table.**

| **Gene name/ Acc. No.** | **dsRNA primers** | **RT-qPCR primers sequences** |
| --- | --- | --- |
| *Dronc* / AAEL011562 | Fw: GGGGTGGTCTTCATCGTG  Rv: GGTCTCCGTATCAGCTTTGG | Fw: AGCTGATACGGAGACCGAAG Rv: GGGTACCGTCGAGAAGCATA |
| *TEP20*/ AAEL001794 | Fw: CGGCATTCGACTGGTTAGC  Rv: TTTATCAAGAAACTCGGTGCG | Fw: CGACCTCTCGCTGTCTACTT Rv: CAACTGGTTTGCTGTCCCAA |
| *Caspase8* / AAEL014348 |  | Fw: CGAATGCCGACTTCCTGTTT  Rv: GCACAACTCTCGGATGAACC |
| *TEP2* / AAEL008607 |  | Fw: CGGCAAGCATCGAGGATAAG  Rv: ATCAGACTTGCCGAAAGCAC |
| *TEP15*/ AAEL014755 |  | Fw: ATCCCAGAAGCCGACAAGAA  Rv: CGACGACAGTGTCCTGAGTA |
| *TEP24*/ AAEL017023 |  | Fw: ATTTCGCTGGTGGAATGACG  Rv: GGGTCCAAACAAATGTCGCT |
| *ATG14* / AAEL001133 |  | Fw: AGTGGTTTCTCGAGCAGTGA  Rv: TTCCCATTTCCTGGCAAAGC |
| *ATG18A* / AAEL013063 |  | Fw: ACGAGGACATACGAATCGCT  Rv: TTGCAGATCTCCGTCCCTTT |
| *Puc* / AAEL010411 | Fw: TACTTAATCACCCGACCGTTG  Rv: CGTGTTTGTTGTCACTTGC | Fw: GCACCAGAACATCAAGCAGT  Rv: CTGATACCGGCTTGACAGTG |
