# Supplementary material for: JNK pathway restricts DENV2, ZIKV and CHIKV infection by activating complement and apoptosis in mosquito salivary glands
Source: PLoS Pathog. 2020 Aug 10;16(8):e1008754. doi: 10.1371/journal.ppat.1008754 (PMC7444518; doi:10.1371/journal.ppat.1008754)
Supplement: S5 Table — (DOCX) [file ppat.1008754.s006.docx]

**S5 Table.**

| **Virus name** | **RT-qPCR primers** |
| --- | --- |
| CHIKV | Fw: AAAGGGCAAACTCAGCTTCAC  Rv: GCCTGGGCTCATCGTTATTC |
| DENV | Fw: CAGGTTATGGCACTGTCACGAT  Rv:  CCATCTGCAGCAACACCATCTC  Probe:/5HEX/CTCTCCGAGAACAGGCCTCGACTTAAA/3BHQ1/ |
| ZIKV | Fw: CCGCTGCCCAACACAAG  Rv: CCACTAACGTTCTTTTGCAGACAT |
