# Supplementary figures and images for: JNK pathway restricts DENV2, ZIKV and CHIKV infection by activating complement and apoptosis in mosquito salivary glands
Source: PLoS Pathog. 2020 Aug 10;16(8):e1008754. doi: 10.1371/journal.ppat.1008754 (PMC7444518; doi:10.1371/journal.ppat.1008754)

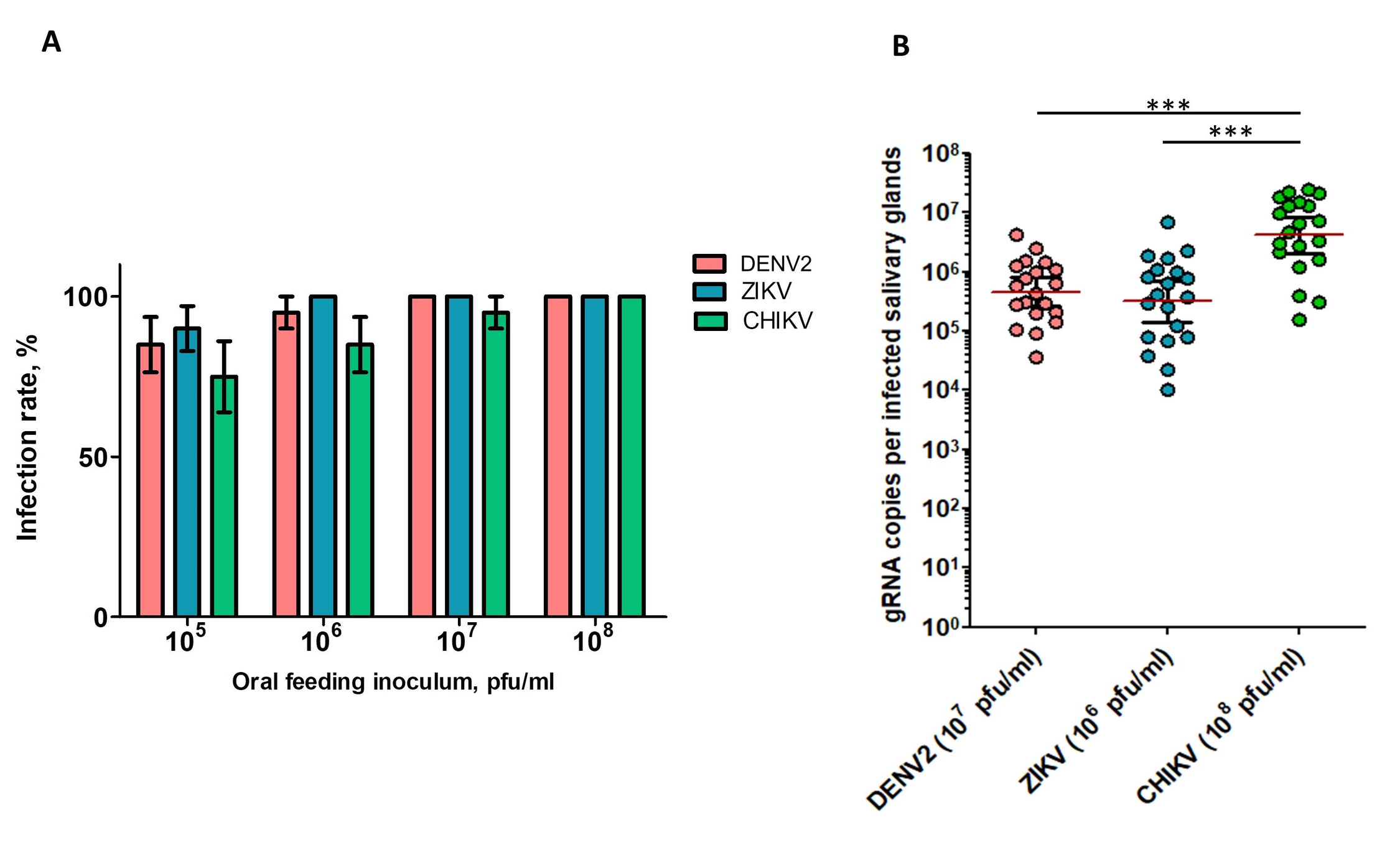

Supplement: S1 Fig — Mosquitoes were orally infected with a blood meal containing 105 to 108 pfu per ml of either DENV2, ZIKV or CHIKV. At 14 days post infection with DENV2 and ZIKV, and 7 days post infection with CHIKV, 20 salivary glands were dissected and virus was quantified using RT-qPCR. (A) Infection rate as measured by the percentage of infected SGs. Bars show percentage ± standard error. (B) Infection intensity as measured by gRNA copies per infected salivary glands. Inocula presented were the one selected for RNAseq. Bars show geometric means ± 95% C.I. (TIF) [file ppat.1008754.s007.tif]

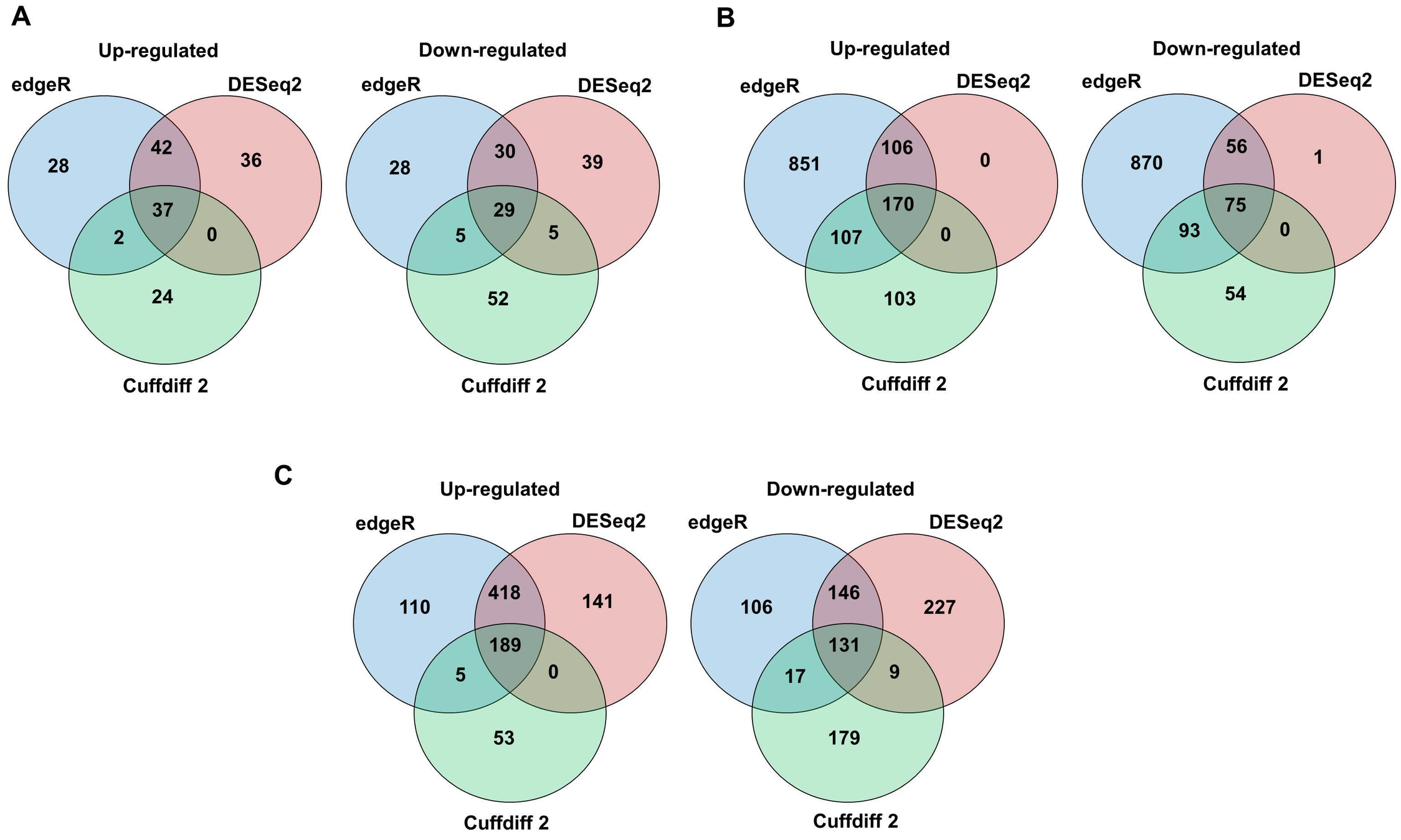

Supplement: S2 Fig — (A-C) Venn diagrams presenting overlaps in up- and downregulated DEGs between edgeR, DESeq2 and Cuffdiff 2 in salivary glands infected with (A) DENV2, (B) ZIKV and (C) CHIKV. (TIF) [file ppat.1008754.s008.tif]

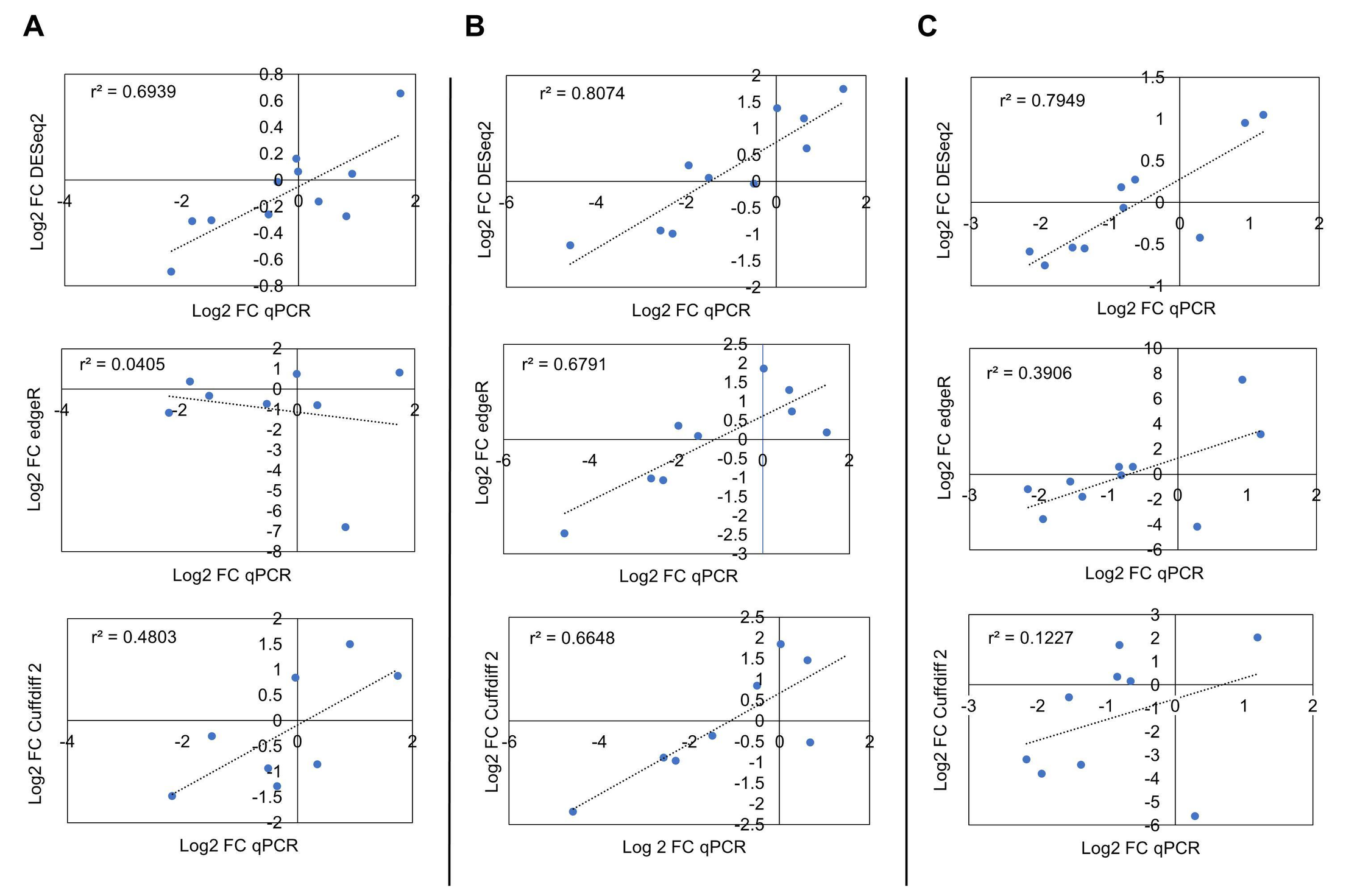

Supplement: S3 Fig — (A-C) The expression of 10 genes in (A) DENV2, (B) ZIKV or (C) CHIKV infected salivary glands was quantified with RT-qPCR and correlated to their respective fold change determined from DESeq2, edgeR or CuffDiff 2 outputs. Log2 Fold-Change (log2FC) is displayed on axes. Three replicates of 10 salivary glands were used for RT-qPCR. RT-qPCR and RNA-seq samples were collected from different biological repeats. r2 indicates Pearson correlation for gene expressions between the two methods. (TIF) [file ppat.1008754.s009.tif]

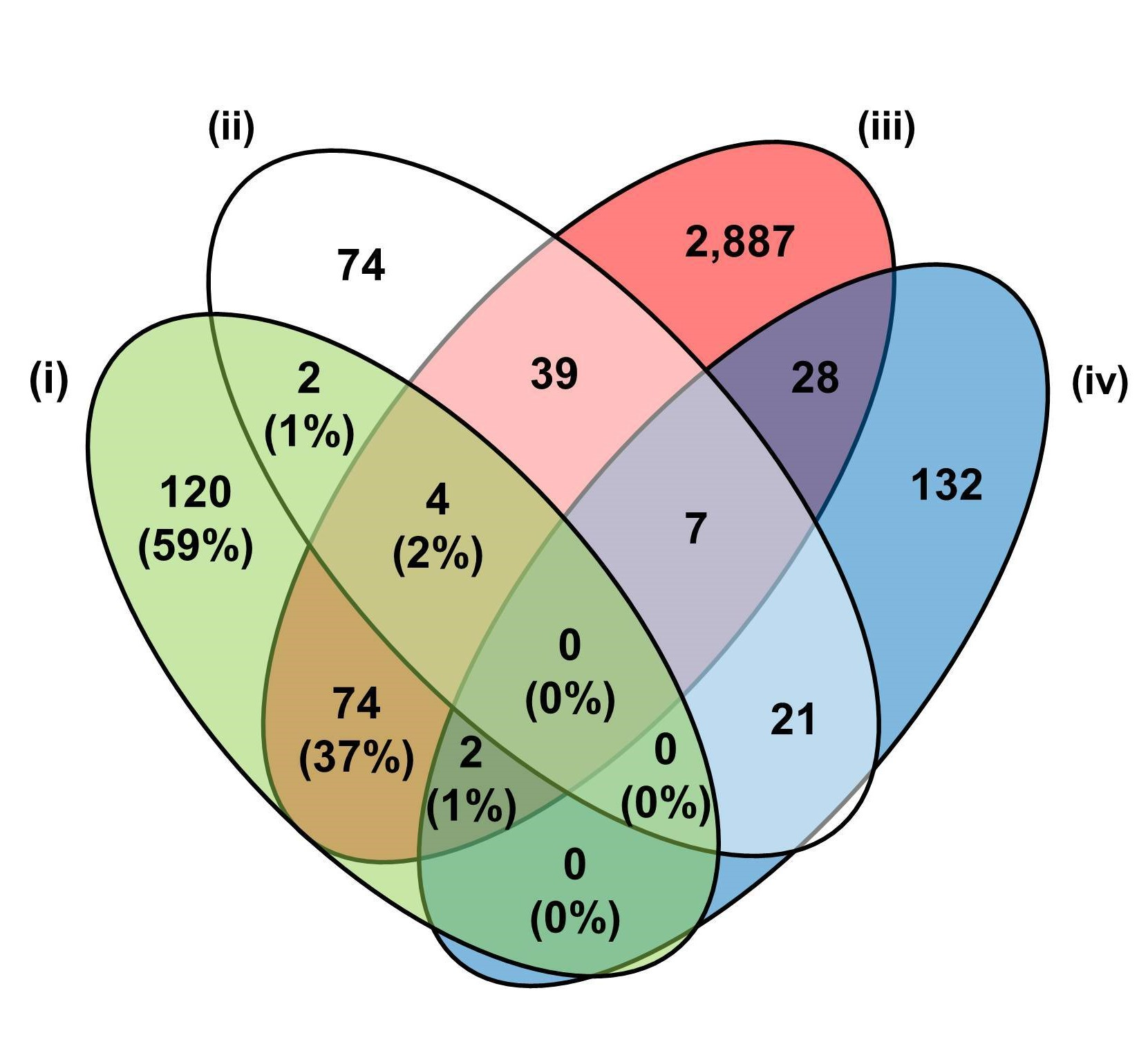

Supplement: S4 Fig — Venn diagram showing common and different DEGs in (i) Singapore A. aegypti colony orally infected with PVP110 virus (this study), (ii) Rockefeller/UGAL A. aegypti colony orally infected with New Guinea C virus [28], (iii) Liverpool A. aegypti colony orally infected with 16681 virus [20], (iv) Chetumal A. aegypti colony orally infected with Jam1409 virus [27]. (TIF) [file ppat.1008754.s010.tif]

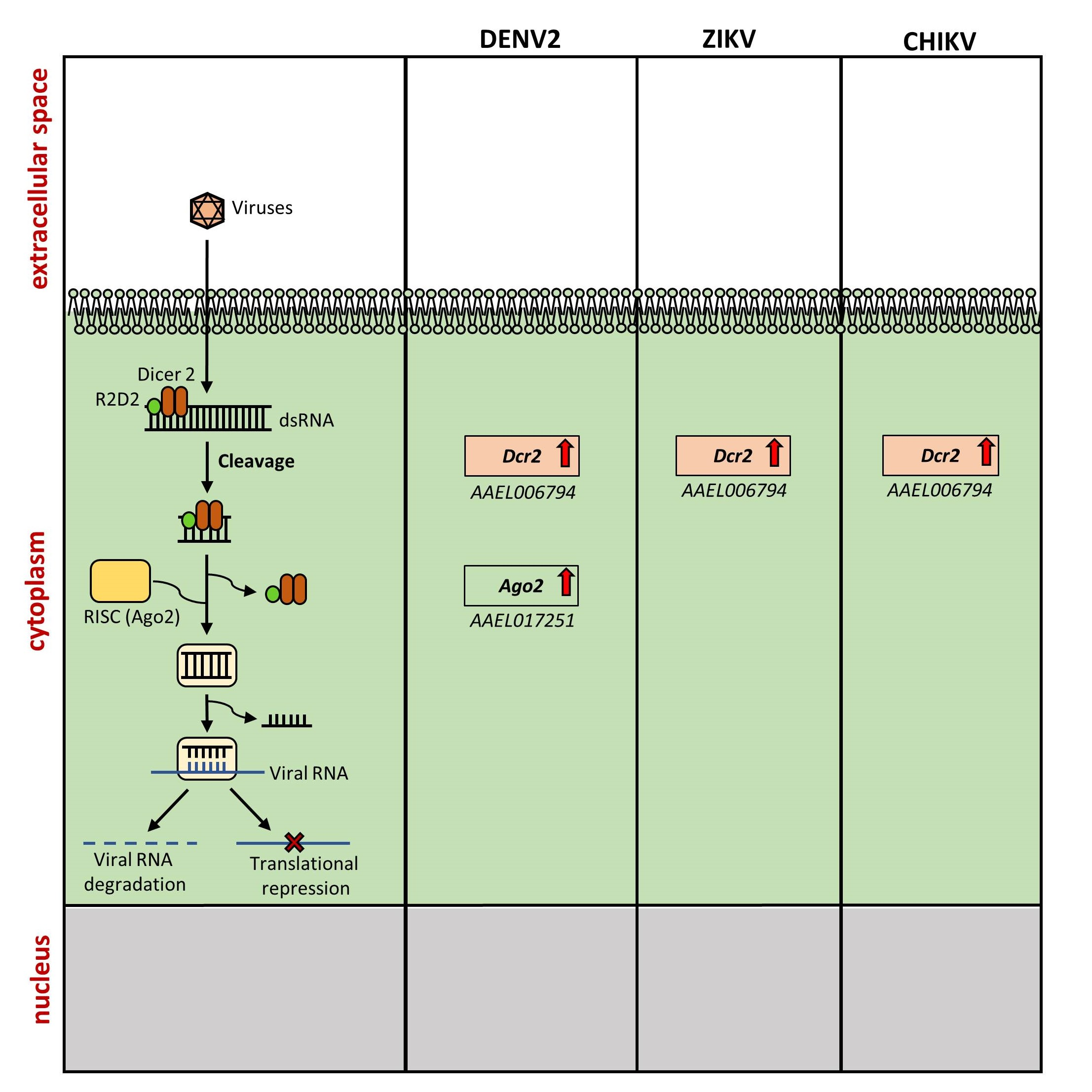

Supplement: S5 Fig — Boxes indicate differentially expressed genes (DEGs) with AAEL number below. Arrows indicate the direction of regulation. Pink boxes indicate DEGs by more than one virus. (TIF) [file ppat.1008754.s011.tif]

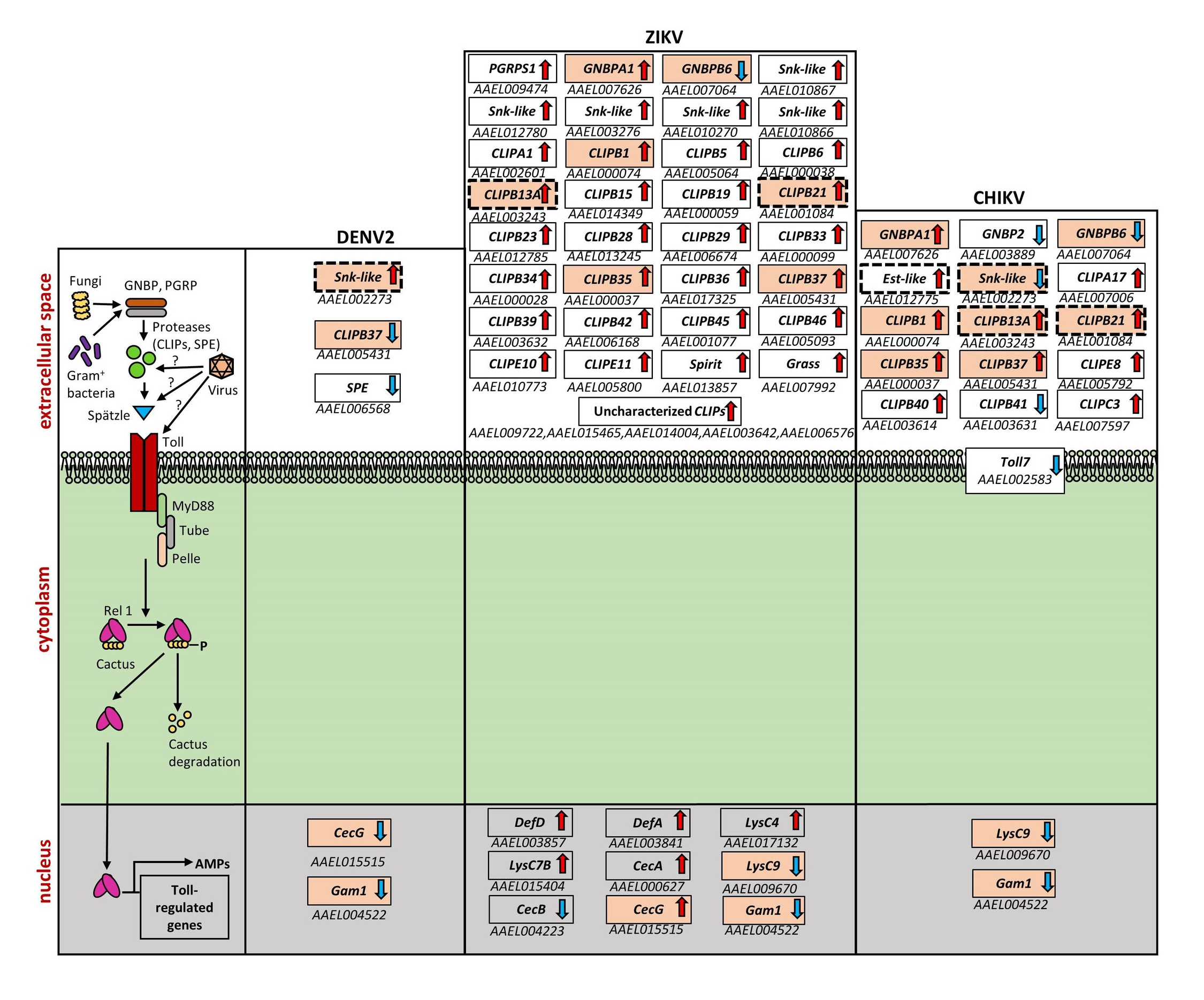

Supplement: S6 Fig — Boxes indicate differentially expressed genes (DEGs) with AAEL number below. Arrows indicate the direction of regulation. Pink boxes indicate DEGs by more than one virus. Dotted boxes show genes selected for functional studies. (TIF) [file ppat.1008754.s012.tif]

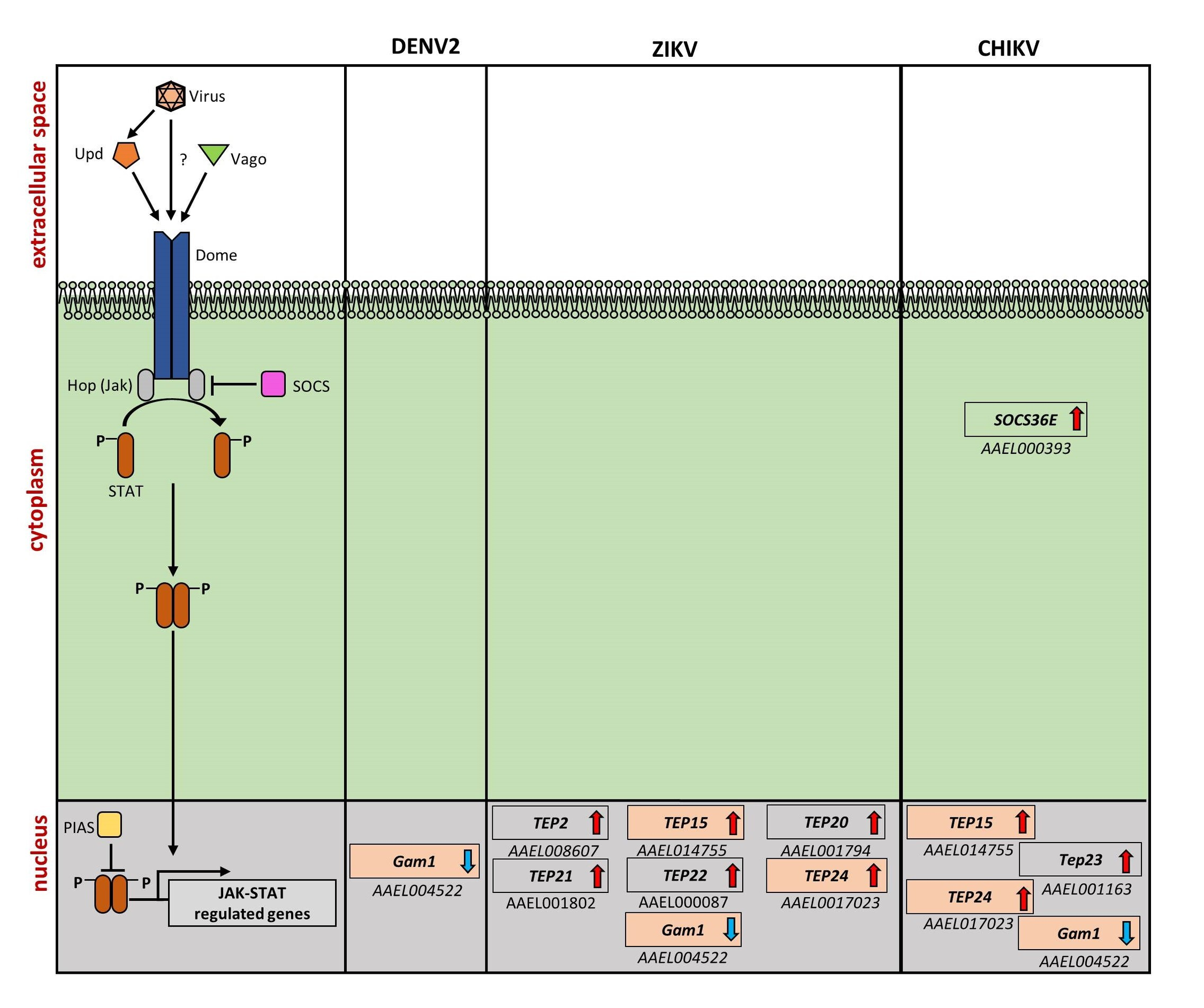

Supplement: S7 Fig — Boxes indicate differentially expressed genes (DEGs) with AAEL number below. Arrows indicate the direction of regulation. Pink boxes indicate DEGs by more than one virus. (TIF) [file ppat.1008754.s013.tif]

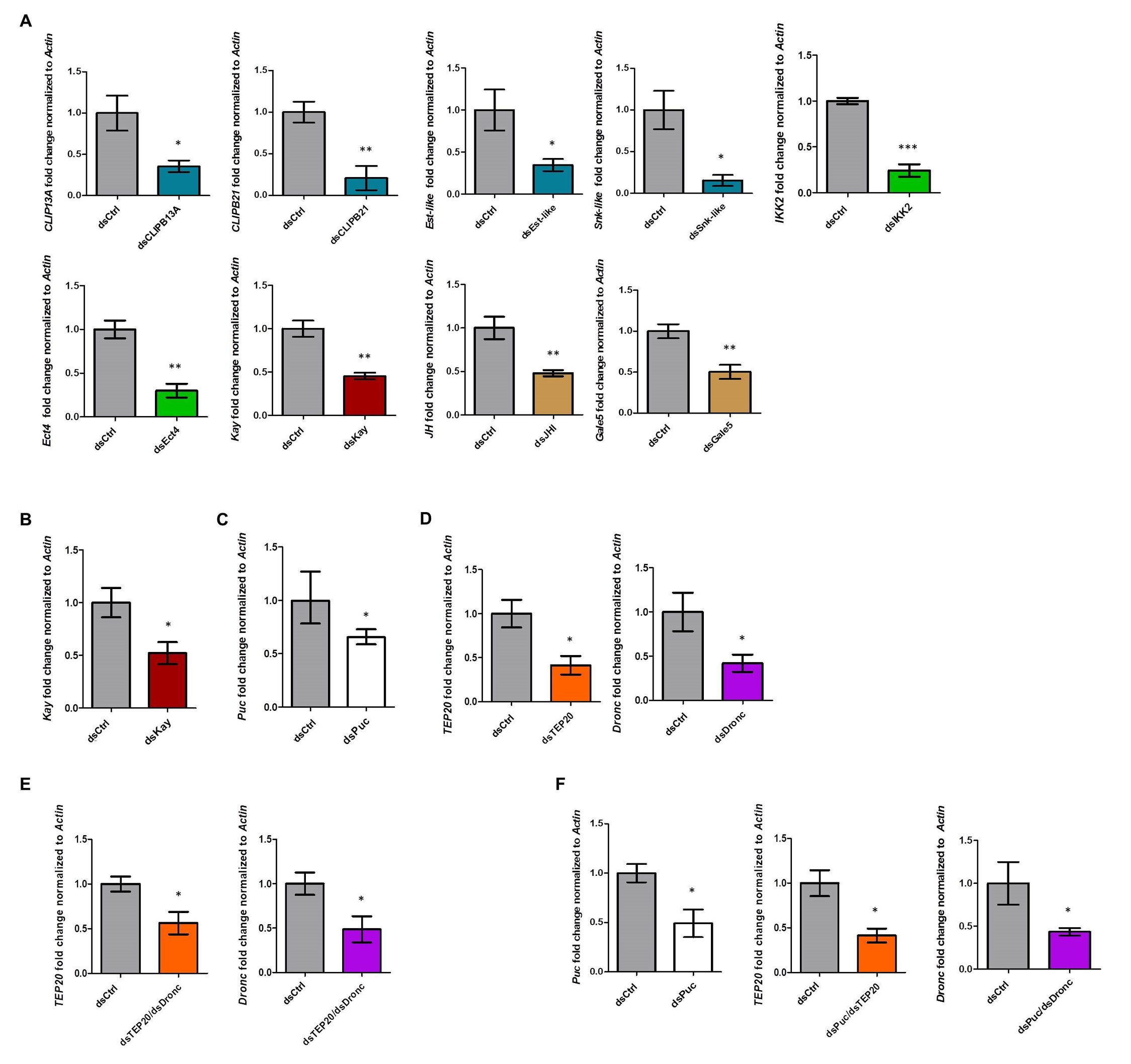

Supplement: S8 Fig — Each mosquito was injected with dsRNA against the candidate gene. Same quantity of dsCtrl was injected as control. Four days later, mRNA was quantified using RT-qPCR in pools of 10 salivary glands or 5 midguts. Actin expression was used for normalization. Corresponding gene expression after injection with (A) 2 μg of dsCLIPB13A, dsCLIPB21, dsEst-like, dsSnk-like, dsIKK2, dsEct4, dsKay, dsJHI or dsGale5 in salivary glands; (B) 2 μg of dsKay in midgut; (C) 2 μg of dsTEP20 or dsDronc in salivary glands; (D) 4 μg of equal amount of dsTEP20 and dsDronc in salivary glands; (E) 2 μg of dsPuc in salivary glands; and (F) 4 μg of dsPuc, or equal amount of dsTEP20 and dsDronc in salivary glands. Bars show means ± s.e.m. from three repeats. dsRNA target: dsCtrl, LacZ; dsCLIPB13A, CLIP domain serine protease 13A; dsCLIPB21, CLIP domain serine protease B21; dsEst-like, Easter-like; dsSnk-like, Snake-like; dsIKK2, Inhibitor of nuclear factor kappa-B kinase; dsKay, Kayak; dsPuc, Puckered; dsEct4, Ectoderm expressed-4; dsJHI, Juvenile hormone inducible; dsGale5, Galectin 5; dsDronc, Dronc; dsTEP20, Thioester containing protein 20. *, p < 0.05; **, p < 0.01, as determined by unpaired t-test. (TIF) [file ppat.1008754.s014.tif]

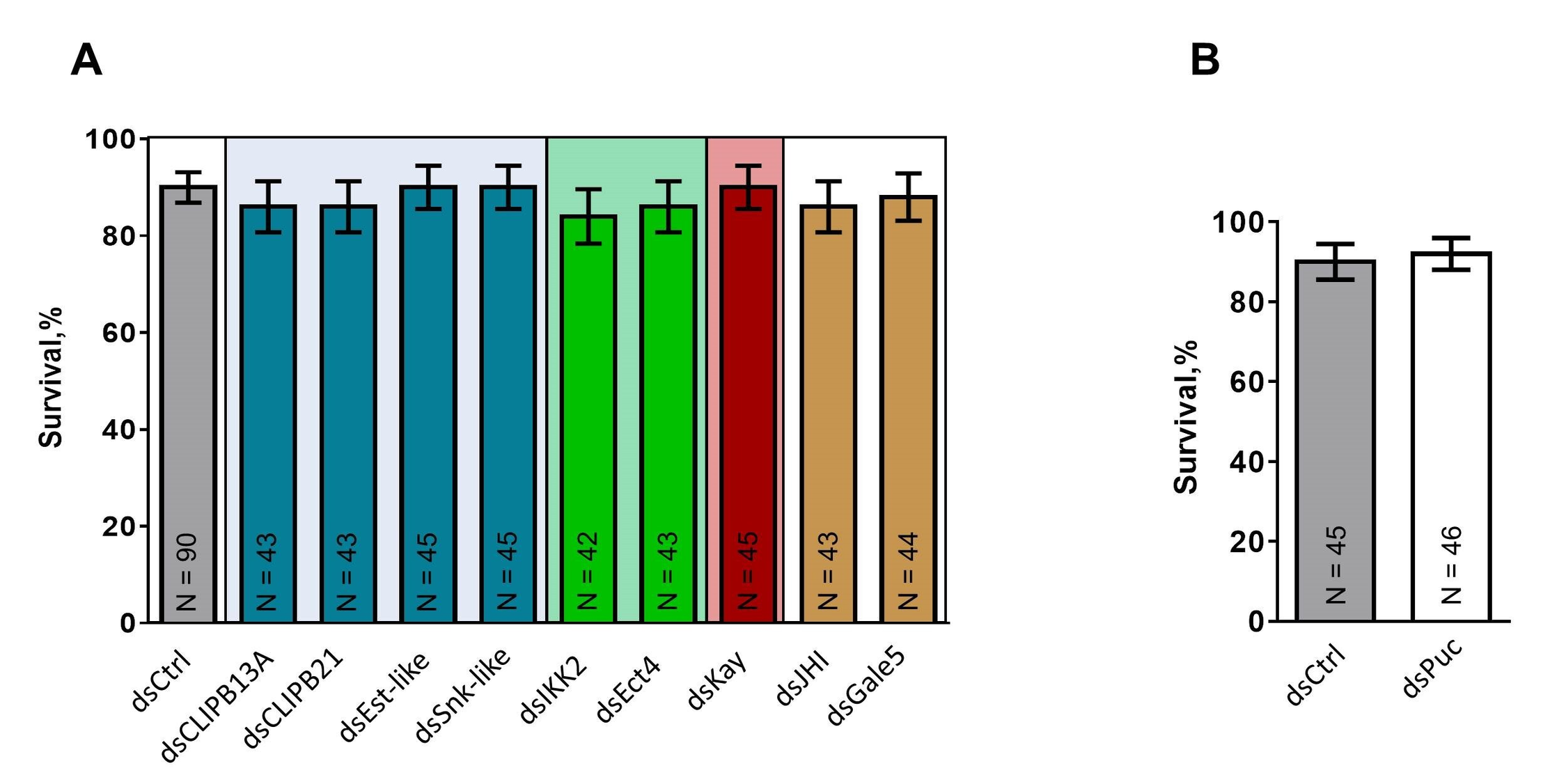

Supplement: S9 Fig — Each mosquito was injected with 2μg of dsRNA against the candidate gene or control dsRNA (dsCtrl). Mosquito survival at 4 days post dsRNA injection against (A) the candidate immune genes and (B) Puckered (Puc). Bars show percentage ± standard error. N, number of dsRNA-injected mosquitoes. (TIF) [file ppat.1008754.s015.tif]

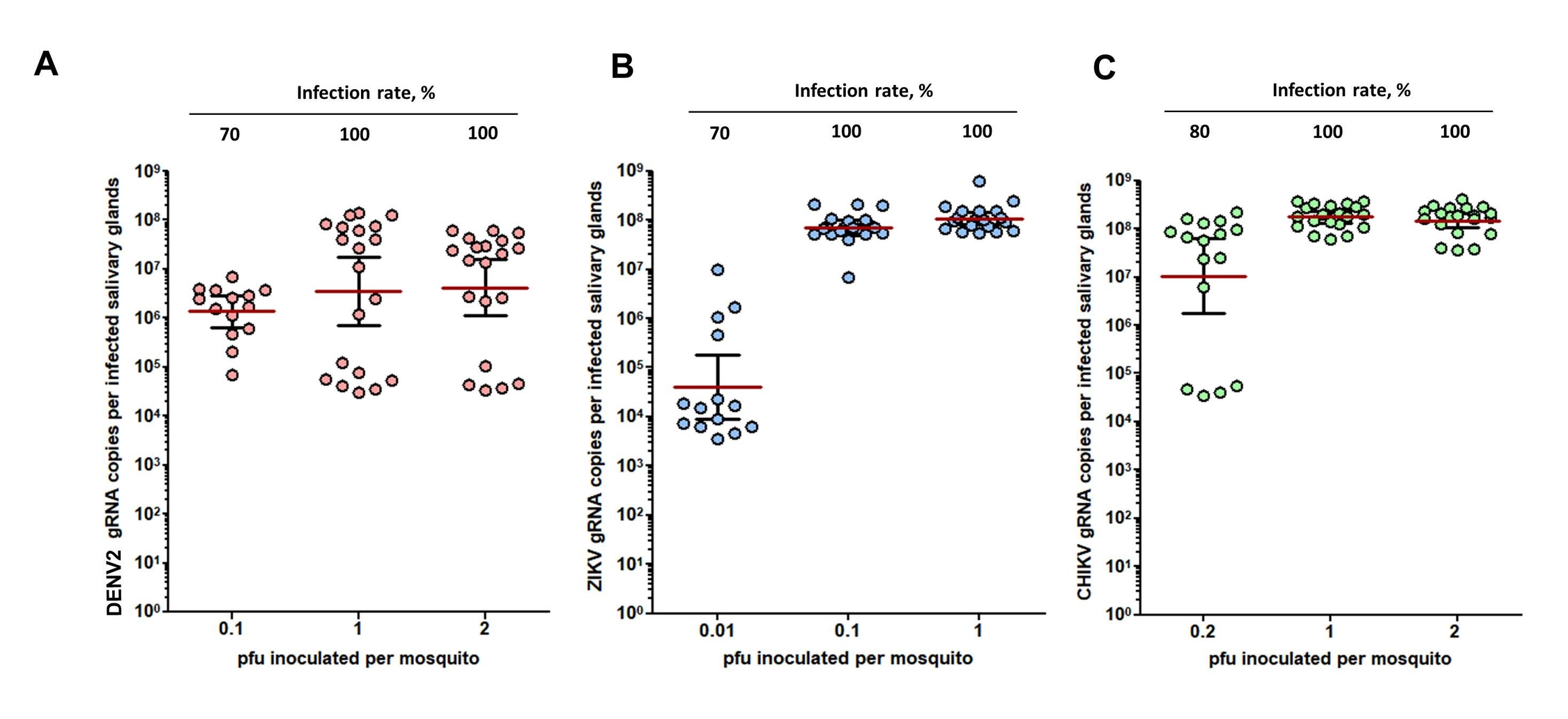

Supplement: S10 Fig — Four days after injection with dsRNA control (dsCtrl), mosquitoes were inoculated with different inoculum doses (plaque forming unit, pfu) of either DENV2, ZIKV or CHIKV. Ten days later, viral genomic RNA (gRNA) was quantified in 20 salivary glands. gRNA copies and infection rate in salivary glands from mosquitoes inoculated with (A) DENV2, (B) ZIKV and (C) CHIKV. Each dot represents one pair of salivary glands. Bars show geometric means ± 96% C.I. (TIF) [file ppat.1008754.s016.tif]

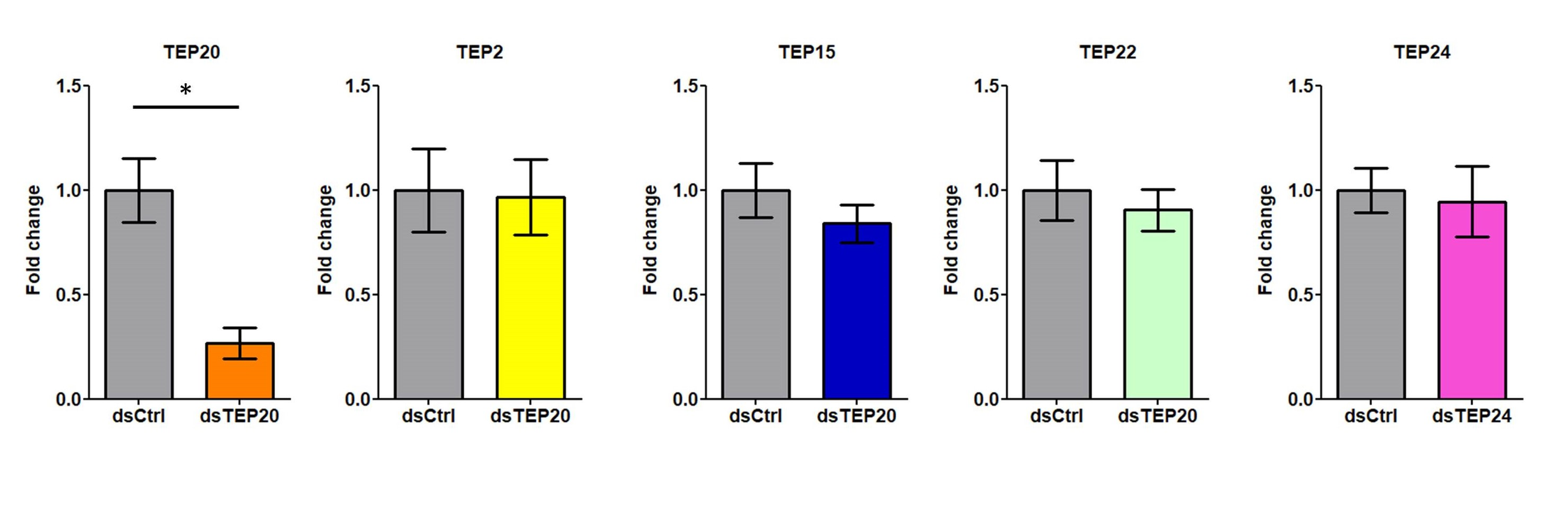

Supplement: S11 Fig — Each mosquito was injected with dsRNA against TEP20 (dsTEP20). Same quantity of dsCtrl was injected as control. Four days later, mRNA was quantified using RT-qPCR in pools of 10 salivary glands. Actin expression was used for normalization. Salivary gland gene expression for TEP20, TEP2, TEP15, TEP22 and TEP24. Bars show arithmetic means ± s.e.m. from three repeats. *, p < 0.05; as determined by unpaired t-test. (TIF) [file ppat.1008754.s017.tif]

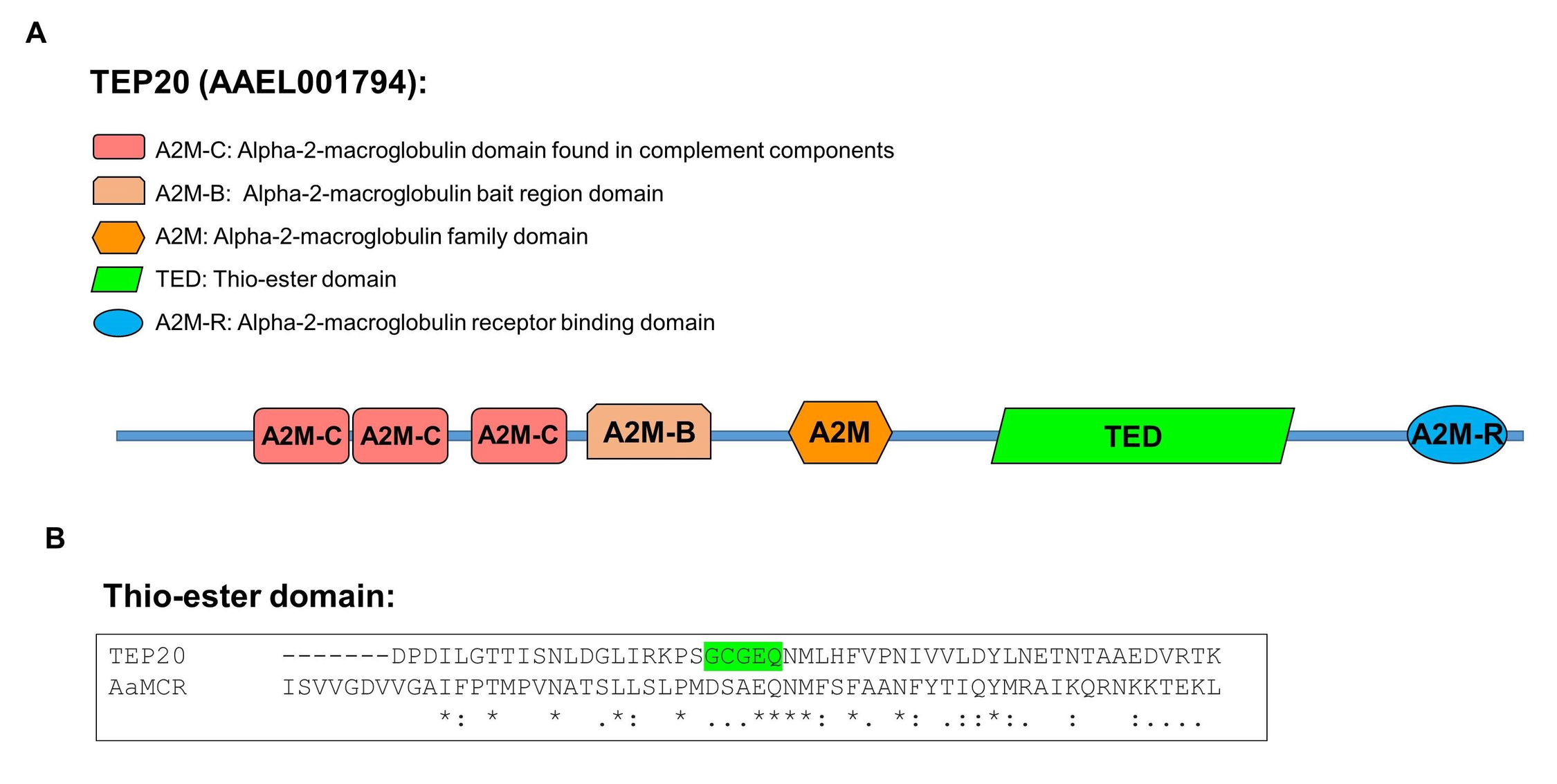

Supplement: S12 Fig — (A) Schematic representation of TEP20 functional domains predicted using the pfam webserver (http://pfam.xfam.org). (B) Localization of the thioester domain in TEP20 and its absence in AaMCR. Sequences were aligned with Clustal Omega (https://www.ebi.ac.uk/Tools/msa/clustalo/)). (TIF) [file ppat.1008754.s018.tif]
