# Supplementary material for: JNK pathway restricts DENV2, ZIKV and CHIKV infection by activating complement and apoptosis in mosquito salivary glands
Source: PLoS Pathog. 2020 Aug 10;16(8):e1008754. doi: 10.1371/journal.ppat.1008754 (PMC7444518; doi:10.1371/journal.ppat.1008754)
Supplement: Attachment — Submitted filename: Response to reviewers_revised#2.docx [file ppat.1008754.s020.docx]

Responses to the reviewers

Dear Editor,

We are thankful to reviewers for accepting our manuscript with minor revisions.

Please find in blue our responses to each comment.

There was no major issues.

**Part III – Minor Issues: Editorial and Data Presentation Modifications**

Reviewer #2: Ln144 and 145, spell out gene names DCR2 and Ago2 for the first time.

The expanded forms have been included in Ln144 and Ln145.

Pg9, Table 1. The color or the RNA-seq based expression induction may be coded with the fold-changes. For instance, the fold-change of “Induced” column could be indicated in the same Table cell.

Log2(fold-change) values for each gene has been indicated in the same ‘induced’ cell as suggested.

Ln 307, Reference Sim et al 2012 should be numbered as other references. Similarly in Ln 312 give the reference number of Sim et al.

The reference number has been included at both Ln 307 and Ln312.

Ln 409, no need to add “the” to Professor

From Ln 409 “the” has been removed.

Figure legends, p-value<0.05 maybe simplified as p<0.05 and so for p<0.01, p<0.001.
Ln 911, S2 Fig. edgerR should be “edgeR”

 “p-value” has been simplified to “p” in all figure legends as suggested.

The spelling error for “edgeR” has been corrected at Ln911.

In addition to the above revisions, we have also done the following minor corrections, which we found upon re-reading. None of the modifications change the result interpretations.

1) Modified sentences in the author summary (from Ln46 - Ln57)
2) Corrected the error in the expanded form of GNBPA1 at Ln148.
3) Corrected the cell ‘induced’ in Table 1 for *Snk-like* (AAEL002273), which is also upregulated by CHIKV, but was mistakenly missing from the earlier version of the table.
4) Corrected a spelling error in Ln256.
5) Corrected the error at Ln284 from ‘7’ to ‘10’ days.
6) Numbered the reference Luplertlop et al,2011 at Ln302.
7) Modified a grammatical error in sentence at Ln344-345.
8) Corrected spelling error at Ln 384.
9) Removed the year of reference Sim et al, 2014 from legend of Figure 1.
10) Corrected reference numbers in S4 Figure legend at Ln927-928.
11) Changed the wrong functional annotations of Myosins/paramyosins from ‘BF’ to ‘CS’ in Table S1.
