# Supplementary material for: JNK pathway restricts DENV2, ZIKV and CHIKV infection by activating complement and apoptosis in mosquito salivary glands
Source: PLoS Pathog. 2020 Aug 10;16(8):e1008754. doi: 10.1371/journal.ppat.1008754 (PMC7444518; doi:10.1371/journal.ppat.1008754)
Supplement: Attachment — Submitted filename: Answers to reviewer comments.docx [file ppat.1008754.s019.docx]

Reviewer's Responses to Questions

Please find our answers to each comments in blue. References to lines were made on the track-changes version.

**Part I - Summary**
Please use this section to discuss strengths/weaknesses of study, novelty/significance, general execution and scholarship.


Reviewer #1: In this study, Chowdhury et al characterized the immune responses of Aedes aegypti salivary glands to dengue, Zika and chikungunya viruses, via both high-throughput transcriptomic assay and RNAi-based functional study. They identified that the JNK pathway, which can be induced by the three viruses infection, may regulate both complement response and apoptosis in salivary glands via TEP20 and Dronc, respectively, to play a common antiviral activity against DENV, ZIKV and CHIKV in Aedes aegypti salivary glands. Generally, this is an interesting study to offer an insight to the antiviral immunity of mosquito salivary glands, which may offer some potential targets to interrupt arboviral transmission. However, the authors should address the following scientific concerns before my positive recommendation.

Reviewer #2: In this manuscript the authors have carried out the experiments with great care and the results are supported by figures in great details. Using RNA-seq authors have done full-genome high-throughput transcriptomic analysis of the Aedes aegypti salivary glands upon Dengue, Zika, and Chikungunya viral infections. This comparative transcriptomic analysis has shown that the major components of Toll, IMD, and JNK pathways were regulated in the salivary glands upon viral infections. Authors have used RNAi-mediated gene silencing to further elucidate the possible mechanisms of JNK-pathway regulated viral defenses in the salivary glands. Finally by expression profiling of the key components of complement pathway and apoptosis in conjunction with the RNAi-mediated gene silencing of positive regulator of JNK pathway or co-silencing of these effectors together with the negative regulator Puckered, authors have concluded that the broad antiviral function of JNK pathway likely derives from the activation of complement and apoptosis pathways in the Aedes salivary glands. The manuscript is in general well prepared, however some of the major conclusions are over-stated. The results are generally clear and well-presented but in my opinion some key experiments are missing from the mechanistic studies of JNK-pathway directed anti-viral defenses that directly impact the novelty aspect of the manuscript. Overall, the transcriptomic analasis and RNAi assays are solid and robust, but at its current stage I feel it lacks the quality for PLoS Pathogens, unless these major and minor comments below are addressed:

We thank both reviewers for acknowledging the interest and the quality of our study. We have answered all comments below and conducted additional experiments to answer their major comments. We are convinced that these additional experiments improved the message of our research article.

**Part II – Major Issues: Key Experiments Required for Acceptance**
Please use this section to detail the key new experiments or modifications of existing experiments that should be absolutely required to validate study conclusions.

Generally, there should be no more than 3 such required experiments or major modifications for a "Major Revision" recommendation. If more than 3 experiments are necessary to validate the study conclusions, then you are encouraged to recommend "Reject".


Reviewer #1: 1. The authors identified TEP20 as a resistant factor against the viral infections. Usually, TEPs play the anti-pathogen activity through a thioester domain. However, some study indicated a TEP-like factor without thioester domain, MCR, resists viral infection by regulating expression of AMPs. Does TEP20 have the thioester domain?

We identified the antiviral function of TEP20 in salivary glands. To answer the comment, we searched TEP20 sequence for the thioester motif. TEP20 possesses the thioester motif unlike AaMCR. This information is now presented in supplementary figure (S12 Fig; shown below) and detailed in the text (**line 281**, “Protein sequence observation….”).

A previous study showed that an antiviral AMP can be induced by DENV in the mosquito salivary glands (Luplertlop et al., 2011). Does TEP20 regulate the AMPs expression in SG?

Based on the cited previous paper, it is an intriguing question. To answer it, we measured the expression of Cecropin A, Defensin A and Defensin D in TEP20-depleted salivary glands at 10 days post ZIKV inoculation. These three AMPs were upregulated by viruses in our transcriptome dataset. We found that TEP20 partially regulates AMP expression as shown in the below figure (Fig 5A): both CecA and Def D were significantly reduced upon TEP20 silencing, while expression of Def A was unchanged. We now present this results in the text and discuss the hypothesis that TEP20 antiviral function depends on its regulation of certain AMPs in mosquito salivary glands (**line 275,** “To test whether TEP20 regulates AMP expressions…”; **line 379,** “In the current study, we show that TEP20 regulates expression of the CecA and DefD…”).

2. There are many TEPs regulated by the viral infections in SG. For functional investigation, the authors silenced the TEP20 gene via dsRNA inoculation. Is it possible that the dsRNA TEP20 causes cross-silencing effect to other TEP genes? How about this silencing specificity?

We thank the reviewer for suggesting this potential technical error. We now present data on the expression of four other TEPs (TEP2, TEP15, TEP22 and TEP24) upon TEP20 silencing in ZIKV-infected SG (S11 Fig; shown below). Our results show that dsTEP20 selectively reduces only TEP20 expression. We added this information in the results (**line 252,** “Because of the high sequence homology among TEPs…”).

3. A co-silencing assay suggests that both TEP20 and Dronc may function in the same antiviral pathway. I just wonder whether knockdown of TEP20 can really regulate the apoptosis in the mosquito salivary glands. The authors should provide more convincing evidence before this conclusion.

To answer this comment, we used TUNEL assay to count apoptotic cells in ZIKV-infected salivary glands upon silencing of TEP20 and Dronc individually, and simultaneously. We injected dsLacZ as control. Number of apoptotic cells were similar in TEP20-silenced and control SGs, whereas it significantly decreased upon silencing of Dronc and co-silencing of TEP20 and Dronc (Fig 5B, C; shown below). This shows that Dronc regulates apoptosis but TEP20 does not.

While other results (Fig 4C) indicated that TEP20 and Dronc may function in the same antiviral pathway in SGs, this added piece of data suggests that TEP20 is not required for apoptosis. We discuss this new result in the manuscript (**line 284,** “To support the role of Dronc in apoptosis…”, **line 388,** “ In our study, we reported…”).

4. Line 145-191, there is too much pathway description in the Results. It is better to remove the most description words to the Discussion.

To clarify our message, we have moved majority of the descriptive text to the discussion and kept the description of the regulated genes for each pathway in the results section.

Reviewer #2: 1) Pg 5 Ln 117, the overlap of the DEGs from SG transcriptomes upon DENV, ZIKV, CHIKV is small, with only 19 DEGs are in common. Could this result be influenced by the infection intensities? Have the authors controlled the infection level, or checked the viral titer and infection prevalence by day 14 in the SGs?

It’s interesting to see in Figure 1 the pathway analysis has suggested the genes been regulated by DENV from IMD and JNK pathways are very small numbers, however there are quite some genes were regulated upon CHIKV infection in the SGs. If JNK is a broad antiviral pathway, I would think similar genes or gene families should be regulated by all these three viruses. I believe it is very essential to measure the infection prevalence and intensities before sample preparations for RNA-seq assays. Authors have provided the information of the viral dosages in the blood meal, however there is no information of the viral titration in the SGs when they have collected samples for RNA-seq. In Figure 1, some of the DEGs were selected for further studies. What are the rationales authors have specifically selected these genes for further functional studies?

We agree with the reviewer that quantification of the infection intensity and infection rate prior RNAseq analysis is critical. Differences in infection intensity may explain the variation in gene regulation as suggested.

In the original version of the manuscript, we presented the infection rate for SG infected with each of the viruses. We used conditions that resulted in 100% infected SG to maximize the transcriptome impact (S1A Fig). This information is now added in the results section (**line 108**, “To maximize the transcriptome impact, we chose virus titers that resulted in 100% infected SGs at the time of collection (S1A Fig)”.

In the revised version, we added the calculation of infection intensity, as determined by the number of gRNA copies in the same samples that were used to estimate infection rate (S1B Fig; shown below). Conditions for CHIKV infection resulted in higher infection intensity that could explain part of the higher transcriptome regulation. This information is now added in the results section (**line 117**…, “Higher transcriptome regulation...”)

Additionally, we quantified the infection intensity using the viral RNA read percentages from our transcriptomic data. Since we used Illumina Ribozero gold kit for ribosomal RNA depletion prior to library preparation, the sequencing data contained reads from both mosquito SGs and viruses. The average ‘percentage of reads’ for each viruses are as follows - 11.16% for DENV, 12.27% for ZIKA and 52.60% for CHIKV. This added piece of data confirms the higher infection intensity for CHIKV than DENV and ZIKV. This information is detailed in the results section (**line117**, “Higher transcriptome….results”).

The rationale to choose the immune genes for further functional studies was provided in the original manuscript. The chosen nine immune genes were upregulated by the viruses and were previously uncharacterized in salivary glands. We chose four genes from Toll pathway (*CLIPB13A, CLIPB21,* *Est-like,* *Snk-like*), two genes from the IMD pathway (*IKK2* and *Ect4*), one gene from the JNK pathway (*Kayak)*, and two putative immune genes - *Galectin-5* (*Gale5*) and a juvenile hormone induced gene (*JHI*). *Gale5* has shown antiviral function against O’nyong-nyong in *A. gambiae*, while juvenile hormone treatment is known to regulate immune gene expression in *D. melanogaster*. Interestingly*, JHI* was upregulated by all three viruses in our study. This can be found in the revised manuscript at **line 167-176.**

2) From RNA-seq based full-genome transcriptomic analysis authors have shown four TEPs are induced upon viral infections in the salivary glands. Furthermore, through qRT-PCR based expression analysis authors have shown that in the Kay gene silenced mosquitoes the expression of TEP20 and Dronc were significantly reduced. Therefore, authors have concluded that upon infection the JNK pathway induces the complement system through TEPs, and apoptosis through Dronc. This mechanistic study of the JNK-mediated antiviral defense in the salivary glands is overstated. Based on the results provided in this study, there is no direct result supporting this conclusion. First of all, authors haven’t provided experimental evidence to support that TEP20 is taking part in the complement pathway. Previous comprehensive study of insect TEPs and Aedes TEP1 protein (Xiao et al., 2014) has concluded that the complement-related protein control the flavivirus infection of Aedes mosquitoes through induction of antimicrobial peptides (AMPs). In that study authors have done phylogenetic analysis of A. aegypti macroglobulin complement-related factors (AsMCRs) and have shown that not all TEPs contain the TE module, and ones lack of this domain have a distinct mechanism of action. In that study, TEP20 was not characterized. In order to establish the link between JNK pathway, anti-viral defense, and complement pathway, authors have to do more detailed studies to show the evidence of TEP20 is indeed a complement-like factor in the Aedes mosquito anti-viral defense. Similar assays done by Xiao et al., 2014 could be applied to TEP20 in this study. Authors should assay the recognition of TEP20 to the tested viruses in vitro and in vivo like AaTEP1(AAEL006361). Another aspect is to study whether the recognition of TEP20 to either one of the tested viruses regulates the expression of AMPs. Without all these direct evidences, the conclusion of JNK pathway restricts viral infection by activating complement pathway in mosquito salivary glands is very vague.

We thank the reviewer for these interesting questions. These also relate to the other reviewer’s issues and most probably will improve our mechanistic description.

We have added several pieces of information to support the role of TEP20.

First, we identified the thioester domain in TEP20 (S12 Fig; shown below). This determines that TEP20 belongs to the subfamily of TEPs with thioester domain. This thioester is used to bind to pathogen surfaces. Demonstration of such interaction would require an antibody, which is unfortunately not available. However, we tested the reported function of TEP in regulating AMPs. We quantified three AMPs that were upregulated upon virus infection in the RNA-seq analysis in TEP20-depleted ZIKV-infected salivary glands. The expression of both CecA and DefD were significantly reduced upon TEP20 silencing, while expression of DefA was unchanged (Fig 5A; shown below). Altogether, these added information improve our understanding of TEP20 antiviral function. However, we believe that a deeper mechanistic characterization of TEP20 function is not the main scope of the paper. We discovered the antiviral role of JNK in salivary glands and determined that it is mediated through TEP20 and Dronc.

We now discuss these data and their implications for TEP20 function in the manuscript. (**line 281,** “ Protein sequence observation…”; **line 275,** “To test whether TEP20 regulates AMP expressions…”; **line 379,** “In the current study, we show that TEP20 regulates expression of the CecA and DefD…”).

3) Similarly, through RNA-seq and RNAi-mediated gene silencing and qRT-PCR assays authors have suggested that JNK pathway restricts DENV, ZIKV, and CHIKV infection by activating apoptosis. Again this conclusion is over-stated. Authors have shown through qRT-PCR that in the Kay gene silenced mosquitoes the expression of Dronc was significantly reduced, however authors jumped immediately to the conclusion that upon infection the JNK pathway induces the apoptosis through Dronc without further direct evidences. I would suggest authors to perform tunnel staining in conjunction with RNAi, qRT-PCR assays to show the direct evidence supporting this hypothesis.

We thank the reviewer for the suggestion and have now performed TUNEL assay on ZIKV-infected SG upon individual or simultaneous silencing of Dronc and TEP20. As depicted in the below figure (Fig 5B, C), Dronc silencing significantly reduced apoptosis when compared to dslacZ (dsCtrl) mosquitoes, indicating the role of Dronc in apoptosis and that JNK pathway activates apoptosis via Dronc.

This information is added in the results and discussion (**line 286,** “Dronc triggers apoptosis by cleaving…”; **line 388** “ In our study, we reported…”).

**Part III – Minor Issues: Editorial and Data Presentation Modifications**
Please use this section for editorial suggestions as well as relatively minor modifications of existing data that would enhance clarity.


Reviewer #1: (No Response)

Reviewer #2: Pg3 Ln63, the citation of Kistler et al., 2015 is not an appropriate reference for refractory mosquitoes.

We thank the reviewer for pointing the inappropriate reference for refractory mosquitoes. In the revised version we have included a correct reference, Jupatanakul et al, 2017. In this article, the authors have generated engineered *Ae. aegypti* with activated JAK-STAT pathway that restricts DENV infection.

Figure 1, Functional annotation of DEGs, the functional group named “BF” seems inappropriate, could some of the genes within this group be classified in the other functional groups, such as “MET”, or “PROT”, or “DIG”, etc?

Mosquito blood feeding is fundamental to disease transmission and mosquito salivary glands are likely a site where the key regulatory genes of blood feeding are produced. For these reasons, we preferred to group ‘Blood-feeding related genes’ that may have or were shown to have a function in blood feeding (including biting). A similar classification of these genes was done in another paper on mosquito salivary glands (Ref: Sim et al, 2012; PLoS Pathogens).

Ln 153, “suggesting activation of RNAi”, this expression is inappropriate, should be “RNAi pathway or RNAi machinery”.

The sentence in the revised article has been modified to include ‘RNAi pathway’ at **line 136**.

Ln154, PGRP and GNBP should be spelled out here when the first time authors have mentioned these molecules

The expanded forms of GNBP and PGRP have been included in the main text at **line 145** and **147,** respectively.

Ln204, the gene silencing efficacy ranged from 35-85% in SGs. The functional studies based on 35% efficacy are irrelevant to the ones with 85% gene silencing efficacy. Authors should either delete the result of the functional studies with this level of gene silencing, or increase the gene silencing efficacy through increasing the dosage of dsRNA.

There is indeed a relatively large variation in silencing efficiency in salivary glands. To ensure that the injected dsRNA reaches the salivary glands, we injected a considerable amount of dsRNA, a total of 2 µg per mosquito for single-gene silencing. To achieve this quantity, we injected 2 drops of 69nl of 14 µg/µl of dsRNA. This strategy is common to other reports (Sim et al. 2012, Anglero-Rodriguez et al. 2017), where significant variation in gene silencing efficiency is also observed. Importantly, gene silencing was evaluated by quantifying mRNA because of lack of antibody. With this in mind, the impact of gene silencing should be interpreted with regards to the impact on the phenotype. In our case, the lowest gene silencing efficiency was for single-gene Puc (S8C Fig), which increased virus load for ZIKV, DENV and CHIKV. The change in virus load indicates that even with a moderate mRNA reduction can have a phenotypical impact.

However, we had mistakenly included the efficiency for Puc when presenting efficiency for the first screen of immune components. In the new version, we mention the variation in silencing efficiency and caution about the interpretation of these with low efficiency (**line 176,** “Variation in gene …”) and removed the 35% to replace with the range (50-85%) of efficiency for the screen on RNAseq regulated immune components.

Fig. 2, It is more appropriate to present the median level of viral titers than the mean ± 95% C.I., considering the distribution of viral loads is not a normal distribution. The Mann-Whitney test and Chi-squared test should be used for the statistical analysis on the infection intensities and infection rate respectively.

We believe there is a misunderstanding and excuse for it. It is exact that the viral load does not follow a normal distribution but a logarithmic distribution. We then log-transformed the data to fit a normal distribution as determined by D’Agostino-Pearson Omnibus (K2, Prism v8). Using this log-transformed normal data, we were able to use parametric tests such as t-test. For infection rate, we use a Z-test, which is identical to Chi² test. Concerning the mean, there is also a misunderstanding. We did not calculate the arithmetic mean for viral load but the geometric mean. The geometric mean indicates the central tendency of a set of numbers by using the product of their values. Mathematically, the geometric mean calculates the arithmetic mean of the log-transformed data. Given that viral loads are log-distributed, we consider that geometric mean is the appropriate measure to compare them. We now clarify our choice of geometric mean and statistical analysis in more detail in the method section (paragraph starting at **line 570**, “In figures, the geometric mean of…”).

Ln 457, label DENV serotype 2 as DENV2 is better to differentiate from other serotypes.

We changed DENV to DENV2 in the entire manuscript.

Ln 470, mosquitoes were left to feed for 1.5 h, is that too long to feed viral infected blood meal to mosquitoes?

Mosquitoes were fed through a membrane feeding system that was filled with blood previously maintained at 4ᵒC. Once the feeder is attached to the membrane feeding system, it takes time to warm up the blood meal to 37ᵒC, which is required to attract the mosquitoes. Therefore, in our insectary, we have optimized the time to maximize mosquito feeding rate to 1.5 h. We now clarify this point in the methods (**line 460,** “The relative long feeding …”).

Ln 472, the viral titers in the blood meal is very different. Does this cause the different expression profiles of immune genes in the SGs through RNA-seq analysis? I would suggest authors to measure the viral loads in the SGs when they did the RNA-seq assays.

We thank the reviewer for this suggestion and now provides the viral load in SGs for the different titer used in RNAseq. We used two methods to calculate it: (i) RT-qPCR on samples used to optimize RNAseq conditions (S1B Fig; shown below) and (ii) virus reads percentage from the RNAseq data (11.16% for DENV, 12.27% for ZIKA and 52.60% for CHIKV). CHIKV viral load is higher. We now present this data and comment on the impact that this difference can have on gene expression variation (**line 117,** “Higher transcriptome regulation...”).

Ln 473, the titration of the viruses in the cell line cultures were measured with plaque assay using BHK-21 cells. However, for the functional studies the quantification of virus genome RNA copies was measured using RT-PCR. It is known viral loa by RT-PCR doesn’t correlate with plaque assay results, and the viral loads estimated with the qRT-PCR method were on average much higher than the PFU levels. Besides the qRT-PCR based assays cannot distinguish among viable virus and gRNA template. Therefore I would suggest authors to titrate the viral loads through plaque assays.

To answer the comment, we generated the mosquito material to perform plaque assay. Unfortunately, the laboratory was locked down on the week we were to perform plaque assay. We propose to conduct these experiments as soon as the lab reopens and add them as an amendment to the paper.

PLOS authors have the option to publish the peer review history of their article ([what does this mean?](https://journals.plos.org/plospathogens/s/editorial-and-peer-review-process#loc-peer-review-history)). If published, this will include your full peer review and any attached files.


If you choose “no”, your identity will remain anonymous but your review may still be made public.


**Do you want your identity to be public for this peer review?** For information about this choice, including consent withdrawal, please see our [Privacy Policy](https://www.plos.org/privacy-policy).

Reviewer #1: No

Reviewer #2: No
